# Waveguide-Enhanced Nanoplasmonic Biosensor for Ultrasensitive and Rapid DNA Detection

**DOI:** 10.3390/mi15091169

**Published:** 2024-09-21

**Authors:** Devesh Barshilia, Akhil Chandrakanth Komaram, Lai-Kwan Chau, Guo-En Chang

**Affiliations:** 1 Department of Mechanical Engineering and Advanced Institute of Manufacturing with High-Tech Innovations, National Chung Cheng University, Chiayi 621301, Taiwan; barshiliadevesh@gmail.com; 2 Department of Chemistry and Biochemistry and Center for Nano Bio-Detection, National Chung Cheng University, Chiayi 621301, Taiwan; akhilsnm7@gmail.com

**Keywords:** optical biosensor, planar waveguide, DNA, real time detection, gold nanoparticle

## Abstract

DNA is fundamental for storing and transmitting genetic information. Analyzing DNA or RNA base sequences enables the identification of genetic disorders, monitoring gene expression, and detecting pathogens. Traditional detection techniques like polymerase chain reaction (PCR) and next-generation sequencing (NGS) have limitations, including complexity, high cost, and the need for advanced computational skills. Therefore, there is a significant demand for enzyme-free and amplification-free strategies for rapid, low-cost, and sensitive DNA detection. DNA biosensors, especially those utilizing plasmonic nanomaterials, offer a promising solution. This study introduces a novel DNA-functionalized waveguide-enhanced nanoplasmonic optofluidic biosensor using a nanogold-linked sorbent assay for enzyme-free and amplification-free DNA detection. Integrating plasmonic gold nanoparticles (AuNPs) with a glass planar waveguide (WG) and a microfluidic channel, fabricated through cost-effective, vacuum-free methods, the biosensor achieves specific detection of complementary target DNA sequences. Utilizing a sandwich architecture, AuNPs labeled with detection DNA probes enhance sensitivity by altering evanescent wave distribution and inducing plasmon resonance modes. The biosensor demonstrated exceptional performance in DNA detection, achieving a limit of detection (LOD) of 33.1 fg/mL (4.36 fM) with a rapid response time of approximately 8 min. This ultrasensitive, rapid, and cost-effective biosensor exhibits minimal background nonspecific adsorption, making it highly suitable for clinical applications and early disease diagnosis. The innovative design and fabrication processes offer significant advantages for mass production, presenting a viable tool for precise disease diagnostics and improved clinical outcomes.

## 1. Introduction

DNA is the fundamental molecule responsible for storing and transmitting genetic information across generations. Analyzing the base sequences of DNA or RNA chains presents vast opportunities for various applications, such as identifying genetic disorders, monitoring gene expression, tracking DNA methylation, evaluating medical treatments, detecting pathogens, and verifying food authenticity [1,2,3]. DNA has also become a cornerstone biomaterial in nanotechnology and nanoscience [4]. The detection of specific DNA sequences is of paramount importance across various domains, including clinical diagnostics, environmental monitoring, horticulture, and food analysis. The ability to analyze gene sequences and genetic mutations offers the possibility of reliable diagnoses even before the onset of disease symptoms. In the environmental and food safety sectors, identifying specific DNA sequences is essential for detecting pathogenic bacteria, fungi, or genetically modified organisms (GMOs). While numerous DNA detection techniques have been developed, polymerase chain reaction (PCR) methods are widely used for their effectiveness in detecting small quantities of DNA. However, the PCR methods have disadvantages regarding the complicated, expensive, time-consuming, and labor-intensive procedures. DNA analysis is also possible through next-generation sequencing (NGS) [5]. This technology is useful in the discovery of new DNA but is expensive and requires advanced computational expertise. Thus, there is huge demand for alternative enzyme-free and amplification-free strategies to allow for ultrasensitive, rapid, low-cost, and user-friendly detection. To this end, DNA biosensors, especially optical DNA biosensors based on plasmonic nanomaterials, are promising candidates [6,7,8]. By leveraging the unique optical properties of plasmonic nanomaterials, these biosensors can detect minute concentrations of biomolecules with high sensitivity and specificity [9,10].

Different surface plasmon resonance (SPR), meta-SPR, and localized surface plasmon resonance (LSPR) biosensors have demonstrated the capability of detecting minute concentrations of biomolecules with high sensitivity and specificity [7,8,11,12,13]. Belushkin et al. have presented a portable digital nanoparticle-enhanced plasmonic imager, a significant innovation in sepsis detection. This device utilizes a unique nanoplasmonic imaging mechanism to detect inflammatory biomarkers in blood serum with high sensitivity and accuracy. The device is small and cost-effective and offers rapid detection, with results available in under 15 min, which is crucial for sepsis management. The system’s performance in clinical settings aligns with gold-standard laboratory techniques, making it a valuable tool for early sepsis diagnosis and potentially reducing mortality rates associated with this condition [11]. Liang et al. have explored enhancement by a LSPR sensor for detecting SARS-CoV-2 by integrating molecular dynamics (MD) simulations with affinity maturation of neutralizing antibodies. Using a novel approach, three engineered antibodies have demonstrated significantly improved affinity, leading to lower detection limits for SARS-CoV-2 pseudovirions. The optimized LSPR platform showed heightened sensitivity and simplified detection, outperforming traditional methods without requiring signal amplification [12]. Chen et al. demonstrate a meta-SPR-based imaging system that integrates a LSPR sensing platform with various microfluidic systems and utilizes simple bright-field imaging. This system offers enhanced detection of biological analytes, enabling the analysis of low-level analyte concentrations from 100 pm to 100 nm. It also facilitates real-time removal of nonspecific binding signals within the same device’s field of view [13].

Waveguides are thin films, cylindrical fibers, or capillaries that efficiently conduct light and have been demonstrated to enhance the sensitivity of many kinds of biosensors [14,15,16,17,18,19,20]. Refractometric optical biosensors using guided-wave technology exploit the phase change caused by the product of a change in local refractive index (RI) near the waveguide surface and the propagation length [21]. Although label-free detection of biomolecules using planar waveguides has been demonstrated [22], the sensitivity is very limited. This is attributed to the long decay length of the evanescent wave, which leads to lower biosensing sensitivity [23,24]. Hence, chromophore- or fluorophore-labelled bioprobes are often applied to enhance the biosensing sensitivity [25]. DNA biosensors using fiberoptic transducers with gold nanoparticles (AuNPs) have gained significant interest owing to their straightforward design, exceptional analytical sensitivity, and cost-efficiency for diagnostic purposes [26,27,28,29,30]. Central to harnessing the potential of DNA in biosensing is its immobilization onto plasmonic nanomaterial surfaces [31]. This step is critical for exploiting the unique optical properties of plasmonic nanomaterials as a signaling agent. In this model study, an oligonucleotide probe was employed to act as a biorecognition probe to allow for specific detection of a target DNA with complementary sequence to the probe. The selective attachment of the oligonucleotide probe onto these surfaces can be achieved through modification with functional groups like amines and carboxylates, enhancing specificity and control over the assembly process [32]. Covalent bonds, particularly those formed between thiol groups and noble metals, further augment the stability and durability of these bioconjugates [33].

Patients with β-thalassemia experience anemia and various pathological complications, including splenomegaly, skeletal abnormalities, and growth retardation [5,34]. β-Thalassemia is caused by mutations in the genes that produce the beta-globin subunit of hemoglobin [34]. Among point mutations, the codon 26 (C^26^) mutation genotype has a high prevalence among carriers for β-thalassemia in Southeast Asia [35]. Thus, the detection of the mutated sequence in C^26^ is employed as a model study here to demonstrate the sensitivity of our waveguide-enhanced nanoplasmonic biosensor for DNA detection without the DNA amplification process, enabling rapid and convenient diagnosis for point-of-care applications.

**Figure 1:**
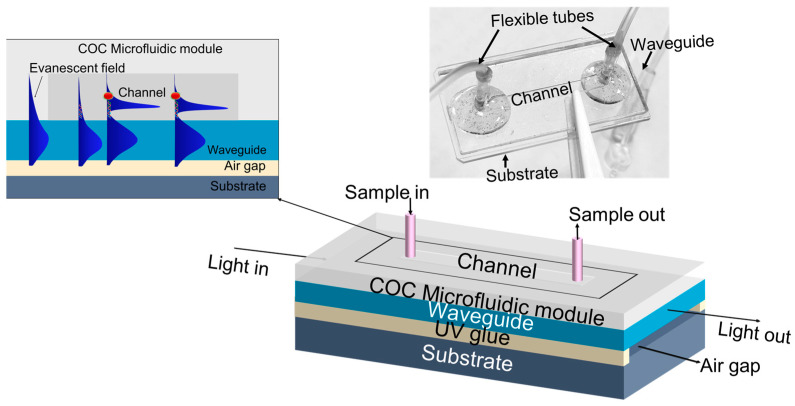
Waveguide biosensor: schematic of the fabricated WG biosensor. Inset: Sensing mechanism and optical image of fabricated device.

In this study, we present an advanced biosensor that incorporates a glass planar WG contrasted with the air gap underneath on a glass substrate, combined with a microfluidic channel fabricated using cost-effective, vacuum-free methods [36,37,38]. The design of the sensor not only minimizes sample volume requirements but also allows for precise manipulation of fluid flow, further enhancing the biosensor’s performance. The entire fabrication process is optimized to be cost-effective and scalable, making it suitable for large-scale production and practical applications. An optical image of the final sensor configuration is presented in the inset of Figure 1, providing a visual representation of the finished device. The novelty of our work lies in the integration of the WG biosensing approach with a nanogold-linked sorbent assay [27,28,39], offering a groundbreaking approach to amplification-free DNA detection. Unlike traditional PCR and NGS methods, we achieved detection of trace amount of DNA using the mutated sequence in codon 26 of β-thalassemia as the target without DNA amplification procedures. We utilized an innovative sensing technique involving AuNPs in a sandwich configuration. The AuNPs in our biosensor perform two key functions: they alter the distribution of the evanescent wave within the WG and induce AuNP plasmon resonance modes, significantly enhancing nanoplasmonic absorption near the DNA target as shown in inset of Figure 1. When light propagates through the WG, an evanescent wave extends into the surrounding medium, decaying exponentially with distance from the WG surface. This decay is characterized by a decay length which is approximately 100 nm under typical conditions. For small analytes like DNA, the decay length is significantly larger than the thickness of the biolayer. This results in only minimal interaction between the evanescent wave and the analyte, leading to a weak response and difficulty in detecting small molecules. However, when AuNPs are introduced, the strong nanoplasmonic resonance significantly alters the evanescent wave’s distribution. The resonance in the presence of AuNPs reduces the decay length to ~76 nm based on the calculation using FEM simulation conducted in COMSOL Multiphysics and localizes the evanescent field near the WG surface. This enhancement allows for the modulation of transmitted light intensity in the WG, enabling ultra-sensitive, highly specific and rapid DNA detection. Our biosensing experiments have shown outstanding performance, achieving an exceptional-low LOD of 33.1 fg/mL (4.36 fM), and a rapid detection in approximately 8 min. The biosensor’s ability to detect this mutation at extremely low concentrations highlights its potential for early diagnosis and monitoring of genetic disorders.

## 2. Experimental

### 2.1. Reagents and Materials

Hydrogen tetrachloroaurate trihydrate (HAuCl_4_·3H_2_O), Tween-20, magnesium chloride (MgCl_2_), sodium hydroxide (NaOH), and sodium dihydrogen phosphate (NaH_2_PO_4)_ were purchased from SHOWA chemicals (Tokyo, Japan). Dextran (70 kDa), carboxymethyl-dextran sodium salt (CMD, 40 kDa), and 3-aminopropyl-triethoxysilance (APTES) were obtained from Tokyo Chemical Industries (Tokyo, Japan). Sodium chloroacetate (ClCH_2_COONa), 1-ethyl-3-(3-dimethylaminopropyl)-carbodiimine hydrochloride (EDC), and N-Hydroxy-succinimide (NHS) were purchased from Sigma Aldrich (St. Louis, MO, USA). Ethanol (99.5%) was obtained from Hy Biocare Chem (Queens, NY, USA). Sodium dihydrogen phosphate (Na_2_HPO_4_) and Tris buffer (2-amino-2-(hydroxymethyl)-1,3-propanediol) were purchased from J.T.Baker (J.T.Baker, NJ, USA). MES buffer (2-(N-morpholino) ethane sulfonic acid) was purchased from Lancaster (Lancaster, UK). Aqueous solutions were prepared using Milli-Q water (Merck, Darmstadt, Germany, resistance 0.054 μS/cm). The oligonucleotides were obtained from Fu Heng Biotechnology Co. Ltd. (Tainan, Taiwan), and their corresponding sequences are shown as follows:

Capture probe (C^C26^) 5′-NH2-C6-AAAAAAAAAAGAAGTTGGTGGTG

Detection probe (D^C26^) 3′-NH2-C7-AAAAAAAAAAACGGGTCCCGGA

Target (T^C26^) TGCCCAGGGCCTCACCACCAACTTC

Negative control (N^C50^) CGGCAGCTGTCCGCGCCCACGGTCCTATGGCGGAGCTAAC GGACTCCGCGCG

### 2.2. Synthesis of Carboxymethyl-Dextran Gold Nanoparticles (CMD-AuNPs)

To prepare CMD, 0.05 g of dextran (70 kDa) and 0.0375 g of sodium chloroacetate were dissolved in 1 mL of 0.5 M NaOH solution. The reaction was carried out for 20 h at 60 °C to ensure thorough carboxymethylation. In parallel, a 0.275 mM HAuCl_4_ solution was prepared by taking 25 mL of the solution and bringing it to a boil under reflux conditions for 20 min. Once the HAuCl_4_ solution had been boiled, 200 μL of the pre-prepared CMD solution was introduced to the HAuCl_4_ solution. The reaction was continued for an additional 30 min, allowing sufficient time for the CMD to effectively interact with the gold ions. After this period, the reaction mixture was cooled down to room temperature. The appearance of a bright ruby-red color in the solution signified the successful formation of CMD-coated AuNPs (CMD-AuNPs).

To purify the CMD-AuNPs, the reaction mixture was centrifuged at 12,580 relative centrifugal force (rcf) and 15 °C for 15 min. This process helped to separate the CMD-AuNPs from any excess unreacted CMD. The supernatant was carefully discarded, which contained the unreacted CMD, and the CMD-AuNP pellet was resuspended in an equal volume of Milli-Q water. This resuspension step was essential to wash and purify the nanoparticles, ensuring the removal of any residual unreacted CMD and resulting in a purified CMD-AuNP solution.

### 2.3. Surface Modification of CMD-AuNPs with Detection Probe (D^C26^)

To conjugate CMD-AuNPs with D^C26^, 4 mL of CMD-AuNPs was mixed with 500 μL of MES buffer (50 mM, pH 6.2) containing Tween-20 (0.1%). Then the mixture was shaken for 10 min to ensure thorough mixing. After this initial mixing, 80 μL of EDC/NHS (32 mM/86 mM) was added in MES buffer to activate the carboxylic (-COOH) groups on the CMD-AuNP surface. Subsequently, this solution was shaken for 30 min at room temperature to allow the activation reaction to proceed. Next, the activated CMD-AuNP solution was centrifuged at 10,000 rcf and 15 °C for 15 min. The supernatant was discarded, and the sediment was resuspended in phosphate buffer (PB) (7.5 mM Na_2_HPO_4_, 2.5 mM NaH_2_PO_4_, pH 7.4) to prepare the solution for the conjugation reaction. Then a solution of D^C26^ was introduced to the activated solution of CMD-AuNPs, ensuring that the final concentration was appropriate for effective conjugation. The reaction was allowed to form an amide bond between CMD-AuNPs and D^C26^ by shaking the solution overnight at 13 °C. After the overnight incubation, the D^C26^-conjugated CMD-AuNPs (AuNP@D^C26^) was centrifuged at 10,000 rcf and 15 °C for 15 min. The sediment was collected and resuspended in 5 mM Tris buffer (pH 8.5) to deactivate any unconjugated active carboxylic sites on the surface of AuNP@D^C26^. Then this solution was shaken for 30 min at room temperature to ensure complete deactivation. Finally, the solution was centrifuged, the supernatant was discarded, and the sediment was resuspended in PB to obtain the purified AuNP@D^C26^.

### 2.4. CMD-AuNPs Characterization

The increased size of CMD-AuNPs was characterized using dynamic light scattering by a Zetasizer nanoparticle analyzer (Malvern Zetasizer Nano ZS90, Worcs, UK). The average diameter of CMD-AuNPs was estimated to be 35.04 nm, while the AuNPs@D^C26^ had an estimated average diameter of 40.73 nm. This increase in size suggests that the immobilization of D^C26^ on the CMD-AuNP surface was successful. The absorbance properties of CMD-AuNPs and AuNPs@D^C26^ were monitored using UV-Vis spectroscopy (JASCO V-570, Tokyo, Japan). CMD-AuNPs exhibited an absorbance of 1.07 a.u. at 520 nm. Upon modification with D^C26^, the absorbance decreased to 1.01 a.u., and the wavelength exhibited a bathochromic shift to 522 nm. These changes in absorbance and wavelength confirm the successful immobilization of D^C26^ on the CMD-AuNP surface, forming AuNPs@D^C26^.

### 2.5. Modification of Glass Planar Waveguide Surface with Capture Probe (C^C26^)

The preparation of glass planar waveguides involved several precise steps to ensure optimal performance as sensing chips. Initially, glass slides (24 mm × 50 mm × 0.2 mm) used as planar waveguides were thoroughly washed with detergent, followed by rinsing with water to remove any residual contaminants. They were then dried overnight at 70 °C to eliminate any remaining moisture. After drying, the glass slides underwent a 30-min cleaning process in an oxygen plasma system to ensure a pristine surface. Following the plasma cleaning, the glass slides were integrated into sensing chips by assembling them with cyclic olefin copolymer (COC) flow channels and glass substrates (see Section 2.6). These assembled chips were allowed to dry overnight to ensure proper adhesion and stability. The next step involved modifying the sensing area of the chips. This modification was carried out by passing a solution, which consisted of 0.4% *v*/*v* (3-aminopropyl) triethoxysilane (APTES), 0.1% *v*/*v* acetic acid, and 5% *v*/*v* Milli-Q water in ethanol, through the flow channels. The solution was allowed to interact with the surface for 30 min to facilitate the attachment of APTES on the glass surface. Subsequently, the chips were washed with ethanol and then with water to remove any excess APTES, ensuring a clean and well-modified surface. To further functionalize the surface, the chips were incubated with a mixture of 0.045 g CMD (40 kDa), 0.05 g 1-ethyl-3-(3-dimethylaminopropyl) carbodiimide (EDC), and 0.025 g N-hydroxysuccinimide (NHS) in 50 mM MES buffer (pH 6.2) for 4 h. This step activated the surface, making it ready for the immobilization of biomolecules. After incubation, the chips were washed thoroughly with MES buffer and water to remove any unreacted chemicals. The immobilization process involved adding a solution of C^C26^ in PB to the WG sensing region. This solution was allowed to interact with the surface overnight at 13 °C, ensuring stable attachment of the biomolecules. After immobilization, any unmodified sites on the surface were blocked using 50 mM Tris buffer (pH 8.5) for 30 min to prevent nonspecific binding during detection experiments. The chips were then washed with PB, making them ready for the detection phase. 

### 2.6. Fabrication and Modification of Sensor Chips

**Figure 2:**
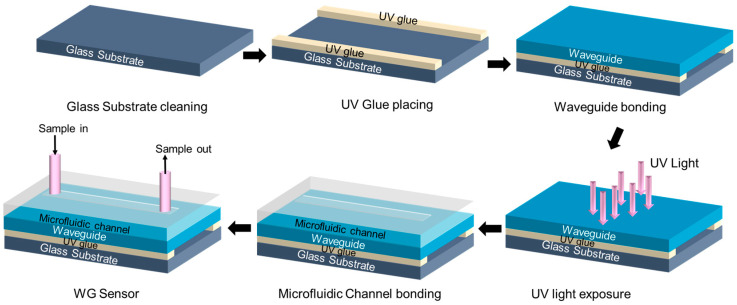
Fabrication processes of sensor chips.

**Figure 3:**
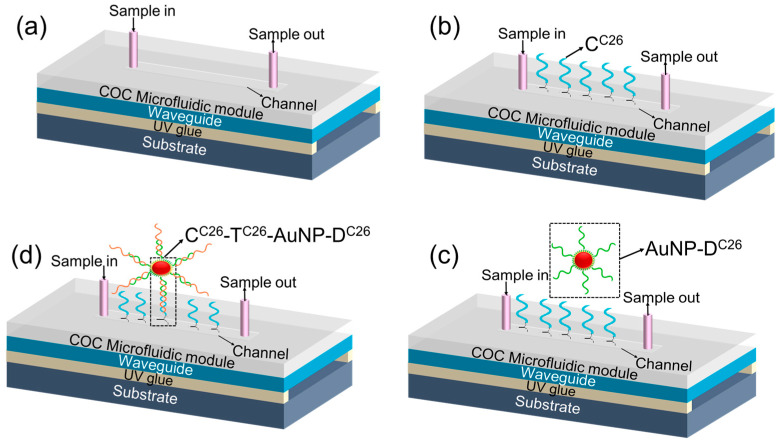
Sandwich nanocomplex architecture. (**a**) Waveguide biosensor chip, (**b**) capture probe (C^C26^) is immobilized on the surface of the WG, (**c**) AuNPs, labeled with a detection probe (D^C26^) to form AuNP bioconjugates (AuNP@D^C26^), and (**d**) sandwich structure (AuNP@D^C26^–T^C26^–C^C26^).

This biosensor is designed with a cover glass (Marienfeld Superior, Paul Marienfeld GmbH & Co. KG, Lauda-Königshofen, Germany), 200 μm in thickness, serving as the planar WG mounted on a glass substrate. The WG structure is designed to guide light efficiently and provides a robust surface for the immobilization of C^C26^. The air gap beneath the WG plays a crucial role in enhancing the interaction between the evanescent field and target DNA molecules, thereby significantly improving the sensor’s sensitivity. The integration of a microfluidic module with the glass WG is another critical design element. This module features a channel that is 3 mm wide, 32 mm long, and 0.2 mm high and is equipped with inlet and outlet tubes positioned at opposite ends. The fabrication processes of the sensor chips are shown in Figure 2 The WG structure is designed to guide light efficiently and provides a robust surface for the immobilization of C^C26^. The air gap beneath the WG plays a crucial role in enhancing the interaction between the evanescent field and target DNA molecules, thereby significantly improving the sensor’s sensitivity. The integration of a microfluidic module with the glass WG is another critical design element. This module features a channel that is 3 mm wide, 32 mm long, and 0.2 mm high and is equipped with inlet and outlet tubes positioned at opposite ends. These tubes are designed to facilitate the controlled delivery of samples to the sensing area, ensuring precise and efficient fluid handling. A 25.4 × 52 × 1 mm glass slide served as the substrate. The fabrication process begins with washing the WG and microfluidic channel with Milli-Q water, followed by drying with a nitrogen gun. Subsequent treatment with oxygen plasma for 30 min enhances the surface properties of the glass, making it more suitable for bonding. After plasma treatment, the substrate was baked at 100 °C for 1 h to further prepare it for the attachment of the planar WG. A 24 × 50 × 0.2 mm glass slide was then cleaned, glued to the opposite side of the substrate. The bonding was completed using UV irradiation for 15 min, which hardens the adhesive and secures the WG in place. The microfluidic channels (32 × 3 × 0.2 mm) were prepared using injection molding, allowing for precise control over the channel dimensions. Flexible tubes were attached to the channels to facilitate fluid injection as shown in Figure 3a. To enhance the sensitivity of detection of specific DNA sequence, the fabricated sensor employs a unique sandwich architecture. Capture probe (C^C26^) are immobilized on the surface of the WG designed as shown in Figure 3b to specifically bind to the target DNA sequence (T^C26^). AuNPs, labeled with a detection probe (D^C26^) to form AuNP bioconjugates (AuNP@D^C26^) as shown in Figure 3c, are then introduced into the biosensor along with the target DNA analyte (T^C26^). The target DNA will hybridize with D^C26^ on the AuNP surface, forming AuNP@D^C26^–T^C26^ nanocomplexes, which are then captured by the immobilized C^C26^ on the WG surface, forming a sandwich structure (AuNP@D^C26^–T^C26^–C^C26^), as shown in Figure 3d. The choice of a longer sensing length is strategic, as it increases the interaction length of light with the AuNP@D^C26^–T^C26^–C^C26^ nanocomplexes, thereby significantly enhancing the light-matter interactions at the WG surface and improving the biosensor’s sensitivity. Since the limit of detection (LOD) of this biosensor depends on how many AuNP@D^C26^–T^C26^–C^C26^ nanocomplexes can be reliably detected above the system noise, a longer optical path also means higher number of C^C26^ probes on the sensor surface and hence lower LOD due to the law of mass action.

### 2.7. Optical Detection System

**Figure 4:**
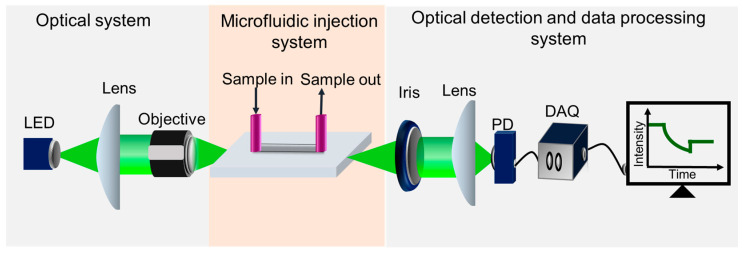
Optical detection system.

The optical detection system designed for real-time measurement of DNA using the proposed biosensor as shown in Figure 4 employs a low-cost green LED (Model 0586-G) with high stability as the probe light source with a power of 3 W. The LED output is modulated by a homemade driver, generating a 1 kHz square wave with a 50% duty cycle. The optical path begins with the collimation of the LED emission using a converging lens. The collimated light is then precisely focused onto the facet of the slab WG through a 20× objective lens. This setup creates an evanescent wave in the sensing region of the WG, which is critical for detecting interactions within the biosensor.

For optimal performance, the sensor is positioned on a 3-axis translational stage. This stage allows for precise alignment and coupling of the light to the sensor chip, ensuring that the light is effectively guided into the waveguide. The transmitted light emerging from the WG’s output facet is filtered by an adjustable iris to eliminate background noise and is subsequently focused on a silicon photodetector. The photodetector converts the intensity of the transmitted light into a photocurrent. The photocurrent is then digitized by an analog-to-digital (A/D) converter and analyzed using a self-developed digital lock-in program to retrieve the signal data. Notably, the LED exhibits a peak emission wavelength of 522.5 nm with a full-width half-maximum (FWHM) of ~30 nm. The plasmonic peak of the AuNPs of diameter 13 nm, following surface functionalization with a self-assembled monolayer, typically aligns at approximately 520 nm. This spectral alignment ensures effective excitation of the nanoplasmonic resonance, thereby facilitating accurate and sensitive detection of DNA.

## 3. Results and Discussion

In this study, we utilized AuNPs with a diameter of approximately 13 nm. AuNPs of this size, when immobilized on a glass substrate, typically exhibit a plasmonic peak around 520 nm [10], which is well aligned with the emission band of our green LED light source. Figure S1 presents the measured LED spectrum alongside the absorption spectrum of AuNPs immobilized on a glass substrate. The LED exhibits a peak emission wavelength at 522.5 nm with a full-width half-maximum (FWHM) of ~30 nm. The plasmonic resonance at this wavelength ensures maximum overlap between the LED emission and the absorption by the AuNPs, resulting in enhanced sensitivity of the detection signal. The purpose of using this optical configuration, as shown in Figure 4, including a narrow-band LED and a photodiode is to simplify the optical configuration without the need of a spectrometer while at the same time achieving high sensitivity, fulfilling the criterion of point-of-care testing.

For a comparison of detection results with and without the labelling of AuNPs on D^C26^, we conducted a FEM simulation using COMSOL Multiphysics. In the presence of AuNPs, the evanescent wave of the probe light interacts directly with the nanoparticles, leading to an energy transfer to the AuNPs, as depicted in the inset of Figure S2. This interaction induces a strong nanoplasmonic resonance, which significantly intensifies the local electric field around the AuNPs and leads to substantial optical absorption, as illustrated in Figure S2. Comparatively, without the AuNPs, the evanescent wave would only interact with the biolayer, resulting in a much weaker signal due to the absence of the plasmonic resonance effect. This pronounced resonance mode significantly increases absorption near the analyte molecule in presence of AuNP, hence confinement within the biolayer. This enhancement is crucial for improving the sensitivity and accuracy of the detection method.

**Figure 5:**
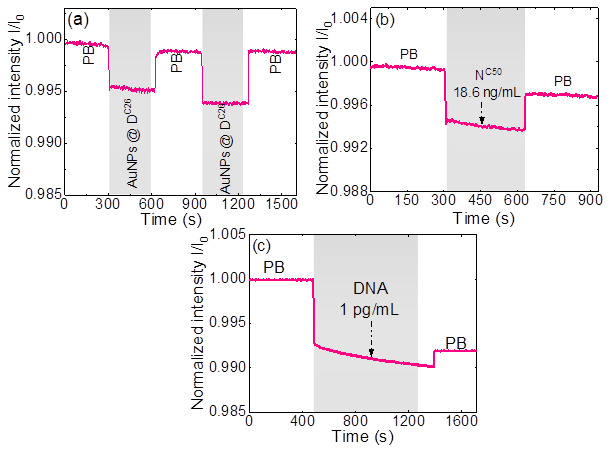
Background nonspecific adsorption test results,(**a**) real-time monitoring results of background nonspecific adsorption tests using blank samples containing AuNP@DC26 but in the absence of TC26(**b**) negative control experiment to check specificity, and (**c**) real-time sensorgram for detection of DNA at 1 pg/mL concentration.

Biosensing experiments were conducted to evaluate the biodetection ability of the devised biosensor. Figure 5a presents the real-time monitoring results of background nonspecific adsorption tests using blank samples containing AuNP@D^C26^ but in the absence of T^C26^, a critical factor that influences the sensitivity, accuracy, and reliability of the biosensor, since nonspecific adsorption of AuNP@D^C26^ on the WG surface in the absence of T^C26^ will lead to a false positive signal. Dextran is a class of neutral and highly hydrophilic polymeric carbohydrates and it has been demonstrated to be a good antifouling material [27,28,40,41]. Therefore, in this study, we employed CMD to coat the WG surface as well as to prepare CMD-AuNPs in order to minimize nonspecific adsorption of AuNP@D^C26^ on the WG surface. The entire experiment was performed using a PB buffer containing 2 mM MgCl_2_ as a binding buffer. Mg^+2^ ions helps to neutralize the ssDNA to allow for hybridization with target probe. Initially, the sensor chip was filled with PB, and the baseline light intensity (I_0_) was recorded. Upon injecting the AuNP@D^C26^ solution, a significant step-shape decrease in transmitted light intensity was observed. This step-shape decrease is attributed to the refractive index (RI) change of the sample solution and light extinction (absorption and scattering) by AuNPs within the evanescent field. Importantly, no biomolecular binding kinetic curve [42] was detected during these injections, indicating that the changes in light intensity were solely due to the solution RI change and nonspecific adsorption of AuNP@D^C26^ on the WG surface and not due to any specific binding interactions. Reintroducing the PB solution restored the light intensity (I_B_) to nearly its original level (I_0_), further confirming the absence of specific binding events. This indicates that RI changes between samples do not interfere with the specific DNA detection experiments. The small difference in signals (ΔI = I_0_ − I_B_) and hence sensor response (ΔI/I_0_) should be attributed to background nonspecific adsorption of AuNP@D^C26^ on the WG surface. This is consistent with the background nonspecific adsorption test results, where two consecutive injections of AuNP@D^C26^ showed sensor response (ΔI/I_0_) changes of 0.000734 and 0.000745, respectively, for the first and second injection. Quantitative analysis of the first injection yielded an average background nonspecific adsorption signal of ΔI/I_0_ = 0.000734 ± 0.000176 across four biosensor chips. This is attributed to the minor nonspecific adsorption of AuNP@D^C26^ on the WG surface. On the other hand, as shown in Figure 5a, the difference in the sensor responses between the first and second injections was only 1.1 × 10^−5^, which is comparable to the system noise’s level of 6.07 × 10^−5^, suggesting that there was negligible background nonspecific adsorption of AuNP@D^C26^ on the WG surface during the second injection. Therefore, we used serial injections of increasing concentrations of T^C26^ at a fixed concentration of AuNP@D^C26^ to construct the calibration curve. To avoid false positive results due to background nonspecific adsorption of AuNP@D^C26^, we set a background adsorption level B = 0.000734 + 3 × 0.000176 = 0.0013 as the cutoff value. Only those sensor responses larger than B were considered positive signals. These results highlight the minimal background nonspecific adsorption, underscoring the biosensor’s reliability and low background signal.

Following the background adsorption tests, we evaluated the biosensor’s performance in detecting DNA. Figure 5c illustrates the sensor’s response to injections of PB as a baseline, followed by a 1 pg/mL DNA standard solution, and subsequent PB injection. The initial baseline with PB established a stable reference light intensity (I_0_). Upon the introduction of the DNA standard solution, a sharp decrease in transmitted light intensity was observed. This intensity drop is attributed to the RI changes and light extinction caused by the AuNPs, which have a substantial extinction coefficient at 520 nm. AuNPs exhibit strong light scattering and absorption, leading to significant changes in light transmission through the WG. This behavior indicates the selective binding of DNA molecules with the functionalized AuNP@D^C26^, forming AuNP@D^C26^–T^C26^–C^C26^ nanocomplexes on the WG surface. The formation of these nanocomplexes significantly alters the local RI and enhances light extinction, resulting in the observed exponential decrease in transmitted light intensity. To remove unbound AuNP@D^C26^ and mitigate light extinction and RI changes caused by free AuNPs in the solution, a PB wash was performed. This washing step restored the light intensity to a certain extent, but it was still significantly lower than I_0_, further confirming that the observed changes were due to specific DNA binding interactions.

**Figure 6:**
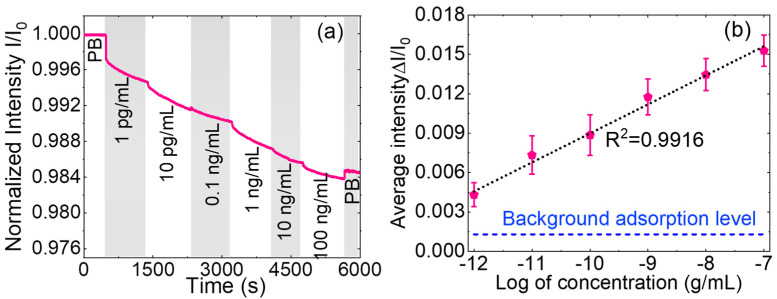
(**a**) DNA real time biosensing for multiple concentrations on single sensor and (**b**) calibration curve.

To quantitatively assess the biosensor’s sensitivity and establish a calibration curve, we tested a range of DNA standard solutions with varying concentrations. Figure 6a illustrates the optical response of the biosensor across a spectrum of DNA concentrations, ranging from 1 pg/mL to 100 ng/mL. The presence of AuNPs in the sandwich structure plays a crucial role in enhancing the detection sensitivity. AuNPs exhibit strong nanoplasmonic resonance when they interact with the evanescent wave generated by the guided light in the WG. This resonance effect enhances the local electric field near the AuNPs, which, in turn, modifies the distribution of the evanescent wave. The result is a reduction in the skin depth of the evanescent wave, concentrating it near the WG surface. This concentration of the evanescent wave near the WG surface leads to several important effects. First, it enhances the field strength, which intensifies the interaction between the evanescent wave and the target biomolecules. Second, it increases optical confinement, which boosts guided wave absorption. These effects collectively result in a significant enhancement of the light–matter interactions at the WG surface, thereby improving the sensitivity of the biosensor. As more target DNA molecules bound to AuNP@D^C26^ are captured on the WG surface, the intensity of transmitted light (I(t)) through the WG decreases. This decrease in transmitted light intensity is directly proportional to the concentration of the target DNA molecules present in the sample [39]. This proportional decrease in light intensity is attributed to the increasing number of AuNPs binding to the WG surface, leading to increasing light extinction by AuNP@D^C26^–T^C26^–C^C26^ on the WG surface. The modulation of the transmitted light intensity allows for real-time monitoring of the target DNA concentration. The biosensor can thus provide ultra-sensitive and rapid DNA detection, with the ability to detect even low concentrations of target DNA at the femtomolar concentration levels.

As the DNA concentration increased, the transmitted light intensity decreased proportionally. Figure 6b displays the calibration curve of normalized average intensity (ΔI/I_0_) against the logarithmic DNA concentration including error bars for N = 3. The error bars represent the variation in results from multiple experiments, calculated as the standard deviation of the mean divided by the square root of the number of experiments conducted. This calibration curve demonstrates the biosensor’s capability for accurate and quantitative detection of DNA. The curve’s high linearity, indicated by the linear correlation coefficient *R*^2^ = 0.9916, underscores the sensor’s reliability and precision in measuring DNA concentrations. Based on the calibration curve, the system’s LOD was calculated using the following equations:

(1) B=a+bx

(2) $$LOD=10^{x}$$

Here, a and b represent the intercept and slope of the calibration curve, respectively. For DNA detection, we achieved an impressive LOD of 33.1 fg/mL (4.36 fM). This impressive LOD highlights the biosensor’s remarkable sensitivity and its suitability for amplification-free detection of trace amounts of DNA using the mutated sequence in codon 26 of β-thalassemia as the target, making it a powerful tool for applications requiring high sensitivity, such as clinical diagnostics. In addition to sensitivity, the biosensor’s rapid detection capability was evaluated by analyzing the biomolecular binding kinetics. The response time of the biosensor, defined as the time required to reach 90% of the equilibrium value, was found to be approximately 8 min. This swift response time is indicative of the biosensor’s efficiency in real-time detection applications, allowing for quick and accurate measurements of target analytes.

A negative control experiment was also performed to test the specificity of the biosensor as shown in Figure 5b. Here a negative control sample containing an unrelated DNA sequence (N^C50^) was used to test the specificity of the D^C26^ and C^C26^ probes. This negative control experiment is crucial to demonstrate that our probes selectively hybridize with the T^C26^ probe and do not interact with non-target ssDNA sequences. As shown in Figure 5b, the sensor response to N^C50^ was negligibly small, having a ΔI/I_0_ change of 0.002571 at an N^C50^ concentration of 18.6 ng/mL. Such a sensor response is smaller than that of T^C26^ even at 1 pg/mL (ΔI/I_0_ = 0.008). This indicates that our biosensor exhibits excellent biological specificity, effectively distinguishing the target DNA from mismatched or non-specific DNA sequences.

The future application of our sensor owing to its high sensitivity and specificity holds significant promise for various analytical applications including clinical, environmental, and food analysis. In clinical areas, the specific detection of DNA has important clinical significance related to diseases of cancer, genetic disorders, and virus infection. In environmental and food areas, the specific detection of DNA can be used for food authentication, the identification of genetically modified organisms, and pathogenic bacteria analysis. These applications highlight the versatility and potential impact of our biosensor in both clinical, agricultural, and research settings. So far, a wide variety of techniques have been developed for amplification-free detection of genetic DNA of β-thalassemia. A comparison of the analytical performance of our biosensor against other available technologies is shown in Table 1, which highlights the superior performance of our device in terms of LOD in comparison to other works:

## 4. Conclusions

In conclusion, this study introduces a novel DNA-functionalized waveguide-enhanced nanogold-linked sorbent assay using an optofluidic biosensor platform for rapid, highly sensitive, and cost-effective DNA detection, crucial for early disease diagnosis. Targeting the mutated sequence in codon 26 of β-thalassemia without relying on DNA amplification techniques, the proposed sensor employs an innovative approach leverages AuNPs to enhance the detection of trace amounts of DNA by modifying the evanescent wave distribution and inducing plasmon resonance. This biosensor utilizes a simple and cost-effective optical setup with disposable chips, requiring only a small sample volume. It features easy miniaturization and low-cost optical configuration. The sensor is easy to fabricate and operate, offering a wide linear dynamic range. It provides an exceptional low limit of detection (LOD) of 33.1 fg/mL (4.36 fM) and delivers a rapid response time of about 8 min. Additionally, it can detect DNA concentrations within a wide linear dynamic range of 5 orders of magnitude, from 1 pg/mL (1.32 × 10^−13^ M) to 100 ng/mL (1.32 × 10^−8^ M), making it ideal for point-of-care testing (POCT).The demonstrated biosensing experiments confirm minimal background signal and high specificity, with the sensor’s performance validated by a high linearity calibration curve. This biosensor demonstrates exceptional sensitivity and rapid detection capabilities, making it a powerful tool for early diagnosis and monitoring of genetic disorders.

## Figures and Tables

**Table 1:** LOD for detection of genetic DNA of β-thalassemia in comparison to other works.

Method	LOD	Linear Dynamic Range	Reference
Quartz crystal microbalance	0.46 nM	2.24 × 10^−6^–2.32 × 10^−9^ M	[43]
Fluorescence	20 pM	5 × 10^−11^–2 × 10^−8^ M	[44]
Magnetoelastic	0.571 pM	1 × 10^−8^–1 × 10^−12^ M	[45]
Electrochemical impedance spectroscopy	35 fM	5 × 10^−13^–4 × 10^−10^ M	[46]
WG-enhanced LSPR biosensor	4.36 fM	1.32 × 10^−13^–1.32 × 10^−8^ M	This work

## Data Availability

The raw data supporting the findings of this study are available from the corresponding author upon reasonable request.

## Supplementary Materials

The following supporting information can be downloaded at: https://www.mdpi.com/article/10.3390/mi15091169/s1, Figure S1: Measured LED spectrum alongside the absorption spectrum of AuNPs immobilized on a glass substrate; Figure S2: Electric field distributions in the sensor with and without AuNPs. Inset: Electric field distribution in vicinity of AuNPs.
