# Supplementary material for: Waveguide-Enhanced Nanoplasmonic Biosensor for Ultrasensitive and Rapid DNA Detection
Source: Micromachines (Basel). 2024 Sep 21;15(9):1169. doi: 10.3390/mi15091169 (PMC11434338; doi:10.3390/mi15091169)
Supplement: Supplementary file 1 [file micromachines-15-01169-s001.zip › micromachines-3189173-supplementary.pdf]

Supplementary Information

Waveguide-Enhanced Nanoplasmonic Biosensor for Ultrasensitive and Rapid DNA Detection

Devesh Barshilia [1], Akhil Chandrakanth Komaram [2], Lai-Kwan Chau [2,*] and Guo-En Chang [1,*]

1 Department of Mechanical Engineering and Advanced Institute of Manufacturing with High-Tech Innovations, National Chung Cheng University, Chiayi 621301, Taiwan; barshiliadevesh@gmail.com
2 Department of Chemistry and Biochemistry and Center for Nano Bio-Detection, National Chung Cheng University, Chiayi 621301, Taiwan; akhilsnm7@gmail.com
* Correspondence: chelkc@ccu.edu.tw (L.-K.C.); imegec@ccu.edu.tw (G.-E.C.)

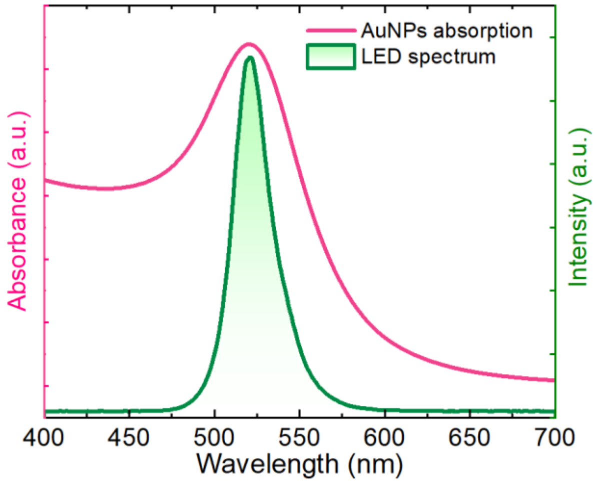

Figure S1. Measured LED spectrum alongside the absorption spectrum of AuNPs immobilized on a glass substrate.

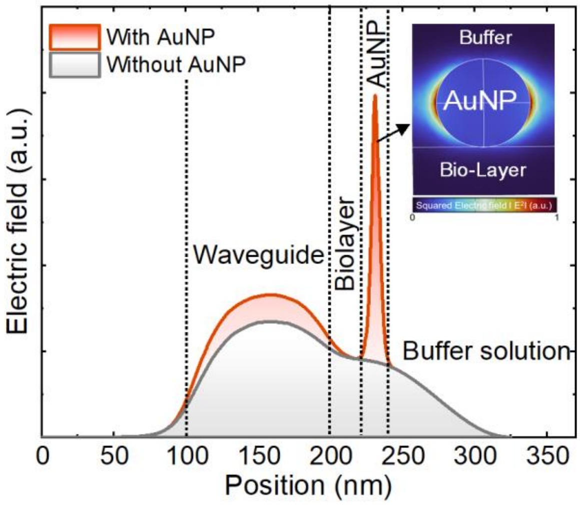

Figure S2. Electric field distributions in the sensor with and without AuNPs. Inset: Electric field distribution in vicinity of AuNPs.
